# Social influence in adolescence as a double-edged sword

**DOI:** 10.1098/rspb.2022.0045

**Published:** 2022-06-29

**Authors:** Lucas Molleman, Simon Ciranka, Wouter van den Bos

**Affiliations:** ^1^ Developmental Psychology, University of Amsterdam, Amsterdam, The Netherlands; ^2^ Social Psychology, Tilburg University, Tilburg, The Netherlands; ^3^ Center for Adaptive Rationality, Max Planck Institute for Human Development, Berlin, Germany

**Keywords:** social learning, social influence, cooperation, belief updating, human development

## Abstract

Social learning is fundamental to human development, helping individuals adapt to changing circumstances and cooperate in groups. During the formative years of adolescence, the social environment shapes people's socio-cognitive skills needed in adulthood. Although peer influence among adolescents is traditionally associated with risky and unruly conduct, with long-term negative effects on educational, economic and health outcomes, recent findings suggest that peers may also have a positive impact. Here, we present a series of experiments with 10–20-year-olds (*n* = 146) showing that positive and negative peer effects reflect a domain-general factor of social information use which declines during adolescence. Exposure to disobedient peers provoked rule breaking, and selfish peers reduced prosocial behaviour, particularly in early adolescence. However, compliant peers also promoted rule compliance and fair peers increased prosociality. A belief formation task further revealed that younger adolescents tend to assimilate social information, while older adolescents prioritize personal views. Our results highlight early adolescence as a key window for peer-based interventions to improve developmental trajectories.

## 1. Introduction

Learning to adequately respond to social information is a central developmental goal, essential for finding one's place in society as an independent and socially responsible adult. Observing the behaviour of others enables people to navigate the diverse interactions they face every day, helping them make socially appropriate decisions and forming accurate beliefs about the world. As children enter adolescence and prepare for adult roles in society, peers become increasingly important sources of social information. Social influence among adolescents is often associated with increased risk taking and negative outcomes (e.g. crime, alcohol abuse, taking drugs and having unprotected sex [1–11]). In the long term, running with the wrong crowd can consequently set people on negative developmental trajectories, with grave implications for health, education, social and economic success, and general well-being [12–14].

Recent research has started to explore the potential of peers to have positive effects on adolescent behaviour, indicating that peers may also reduce risk taking and promote prosociality [15–21]. This suggests that there may be a domain-general mechanism that drives social influence in different decision contexts. However, empirical evidence for this idea is limited because studies of peer influence in adolescence typically focuses on a single behavioural domain at a time, and each study involves its own particular age bracket. As a result, it remains unclear whether positive and negative peer influence on adolescents' behaviour follow the same or different developmental pathways. Furthermore, the research emphasis on risk taking—often measured with hypothetical scenarios and self-reports rather than incentivized behavioural tasks—has led to a negligence of social influence on adolescents’ behaviour in other important domains, such as rule compliance and belief formation. Understanding the development of the behavioural mechanisms of social influence in these domains, and how they relate to each other, is essential for facilitating adolescents' attunement to their social environment, reducing anti-social behaviour and promoting socially desirable outcomes [13].

In this paper, we present evidence from a set of incentivized behavioural experiments with 146 adolescents (ages 10–20, 45% female) targeting three core domains of decision-making: rule compliance, belief formation and prosociality. Our first experiment addresses social influence on rule compliance. The smooth functioning of human societies critically depends on individuals complying with rules that facilitate social coordination and curb the spreading of disorder [22,23]. Developmental studies on risk taking have provided valuable insights in the role of peer influence when adolescents face uncertainty [6,8,21,24–27], but they provide little behavioural insight into rule violations, one of the hallmarks of adolescent behaviour [28]. Our second experiment addresses social influence on belief formation. With the advent of social media, adolescents are continually exposed to social information, which helps acquiring useful knowledge [29] and make accurate decisions [30,31], but can also misinform [32] and fuel group polarization [33]. Yet, although some research has studied belief formation across adolescence [34], the role of social information in this process remains unclear. Our third experiment examines social influence on prosociality, a key building block of cooperation between individuals in society [35]. This experiment allows us to examine the robustness of recent results showing peer influence on adolescents' willingness to help others [20], and further investigate how this relates to individuals’ social information use in other decision contexts. Across all experiments, rather than relying on hypothetical scenarios or self-report, behaviour was incentivized, providing a proper test of peer influence because changing behaviour upon observing peers has real consequences [36].

## 2. Methods

We recruited 146 participants aged 10–20 years from the area of Berlin, Germany, with a balanced distribution across these ages and gender (table 1). We implemented a within-subjects design. Participants completed a series of computerized tasks and questionnaires on their own, in a separate room. All tasks were programmed in LIONESS Lab [37] and are available in editable form via lioness-lab.org. The tasks were administered in the participants' native language (German). At the beginning of each session, participants received general instructions; always from the same test leader, who remained available to answer questions throughout a session, but was seated in a separate room during the session.

**Table 1.** Numbers of participants included in the analyses, broken down by age, gender and experimental tasks. We aimed for a sample balanced across ages, and within each age group, a balanced gender distribution. Note that for some analyses the number of included participants was reduced because of missing outcome variables (see §§2c, 2d and 2e).

age	total	male	female	figure 1		figure 2	figure 3		figure 4
				rule following		belief formation	prosociality		factor *S*
				bad example	good example		selfish example	fair example	
10	16	10	6	9	7	16	15	8	15
11	21	7	14	10	11	21	15	12	19
12	18	10	8	10	8	18	13	12	17
13	9	4	5	6	3	9	5	5	9
14	13	10	3	7	6	13	8	8	11
15	13	9	4	8	5	13	7	11	12
16	13	7	6	7	6	13	5	10	12
17	12	7	5	7	5	12	8	7	12
18	9	5	4	4	5	9	5	8	9
19	12	7	5	3	9	12	11	7	12
20	10	5	5	6	4	10	4	7	10
sum	146	81	65	77	69	146	96	95	138

Tasks were administered in two approximately 60 min sessions, which took place approximately four weeks apart to avoid carry-over effects between sessions with similar tasks (i.e. the two treatments of the task measuring prosociality; see §2c below). To reduce carry-over effects between experiments in a session, task order was randomized within and between sessions, independently between participants. Task-specific instructions were provided on-screen (see section ‘Experimental Procedures and Materials’ in the electronic supplementary material for full experimental materials, translated from German to English).

Tasks were incentivized such that each decision was payoff-relevant, and expected hourly wages were constant across tasks. Instructions for each task concluded with compulsory comprehension questions. After completing the second session, participants received payment in cash (flat fee of €30 plus a performance-specific bonus between €5 and €16). In the instructions of all tasks, peers were introduced as other participants who previously completed the tasks. The information letter for participants pointed out that, in accordance with the rules of the local laboratory, no deception was used in our experiments. To gauge whether participants believed that the peer behaviour they observed was real, we asked participants during debriefing about their general impression of their peers in the experiment, both in writing ('What was your general impression of the previous participants?') and orally by the test leader. We did not find any indication to suggest that participants did not believe the instructions or the veracity of the social information presented to them.

### (a) Rule compliance

**Figure 1.**
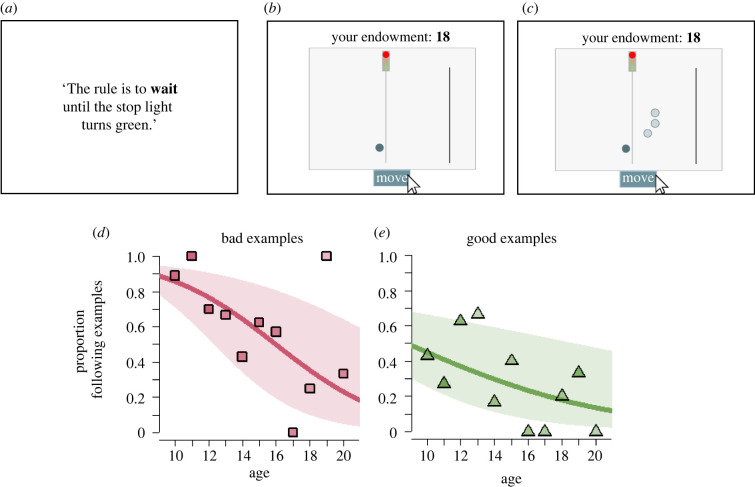
Social influence in rule compliance decreases during adolescence. (*a–c*) Traffic light task measuring social influence in rule compliance. (*a*) Participants were informed that the rule was to wait until the stop light turns green, and were told that rule compliance was against their self-interest. (*b*) Participants first completed the task alone. They could move the circle figure across the screen by clicking the ‘Move’ button. Clicking once made the circle approach the red light and stop to wait. Clicking ‘Move’ again made the circle move across the screen and over the finish line (vertical line on the right-hand side). The timer on top of the screen started at 20 and counting down until participants crossed the finish line. (*c*) Secondly, participants completed the task in the presence of three peers who either violated or complied with the rule. The movement of these peers were based on real previous participants, and were selected such that they always displayed the opposite behaviour than the participant's own first choice (main text). In a third iteration, participants completed the task alone again. (*d,e*) Proportions of participants switching their behaviour when peers were present as a function of age. (*d*) Observing rule violations (shorthand: ‘bad examples’) substantially affect behaviour among younger age groups but peer effects decline across age groups. (*e*) Observing compliant peers ('good examples') has a less pronounced effect. Lines show regression predictions, with standard errors in shades (see 'Methods'). For numbers of observations per data point, table 1. (Online version in colour.)

To measure social influence in rule compliance, we administered a stylized, animated task (adapted from [38]). Participants could choose whether or not to comply with a rule, in the absence and presence of peers. They had to move a circle figure across their screen by clicking a button. In the middle of the screen stood a red traffic light (figure 1). The instructions stated that ‘the rule is to wait until the traffic light turns green’. However, waiting was costly: participants started with an endowment of 20 points (total worth: €1.00), and each second they spent in the task, this endowment was reduced with 1 point until it reached zero. After 12 s, the traffic light turned green. Instructions and control questions made it clear that the rule was not enforced (and that there were no risks of negative consequences for violating the rule), so compliance was voluntary and against one's material self-interest.

Participants completed three iterations of the task, one of which was randomly chosen for payment after the task had completed. In the first iteration, participants completed the task alone. Based on their initial decision, we allocated them to either of two conditions that differed in the displayed movements of three peers who completed the task before. In particular, these peers always chose the opposite of a participant's own initial choice: participants who initially complied with the rule (i.e. moved after the traffic light turned green) were shown three ‘bad’ examples, peers who moved as soon as possible. Participants who initially violated the rule (i.e. moved before the traffic light turned green) were shown three ‘good examples’, peers who waited until the traffic light turned green. This set-up allows us to study age trends in switching in both conditions, but the endogenous treatment allocation limits our ability to directly compare rates of switching across conditions due to selection effects (for example, initial compliance might be correlated with choice consistency). Table 1 details the number of participants allocated to either of these endogenous treatments.

For both conditions, we measured the proportion of participants following social information, switching their behaviour from compliance to violation (or vice versa). An animated gif of the task (German original) without and with peers can be viewed here and here, respectively. The quantity of interest—measuring social influence—was whether or not a participant switched their behaviour upon observing peers choosing the opposite from their own. To test whether social influence effects would spill over into later behaviour, the task concluded with a third iteration in which participants completed the task on their own again.

### (b) Belief formation

**Figure 2.**
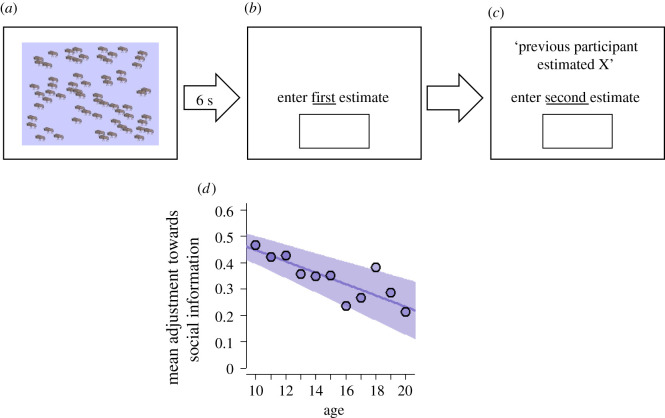
Social influence in belief formation decreases during adolescence. (*a–c*) Perceptual judgement task measuring social information use in belief updating. (*a*) Participants repeatedly observed images showing a number of animals. (*b*) After 6 s, the image disappeared and participants had to estimate how many animals were shown. (*c*) Then, they observed social information (the estimate of a participant who completed the task before), and entered their second estimate. We measured social information use as the extent to which participants adjusted their estimate towards the social information (see 'Methods'). (*d*) Mean adjustments towards social information as a function of age. The line shows the prediction of a regression, with standard errors in shades (see 'Methods'). For numbers of observations per data point, table 1. (Online version in colour.)

To measure how peers impact belief formation, we administered the BEAST (figure 2), a validated task reliably measuring individuals' propensities to integrate social information in perceptual decisions [39], which has been used in adolescent samples [40,41]. The task involves judgements in a context where social values or preferences are irrelevant. For five rounds, participants observed an image with 50–60 animals for 6 s and had to estimate how many there were. After entering their first estimate (*E*_1_), participants observed social information (*X*) in the form of the estimate from another participant who observed the same image. Then, they made a second estimate (*E_2_*).

Social information was selected from a pre-recorded pool of 100 previous participants. We selected an estimate at intermediate distance from a participant's first estimate. In rounds 1–5 of the task, the target deviations of *X* from *E_1_* were 25%, 15%, 20%, 15% and 25%, respectively, and we selected a pre-recorded estimate closest to that target [39]. This allowed for a varying yet relatively constant room for adjustment, without the social information being too far away to be ignored altogether [42]. Social information always pointed in the direction of the true value (*T*), and could therefore be lower or higher than *E_1_*, depending on whether the participant initially over- or underestimated the number of animals. The pre-defined target deviations imply that social information could be farther away from the true value when a participant's first estimate was close to the true value; in those cases, *E_1_* and *X* bracketed *T*. Finally, when *E_1_* was exactly correct, we randomly determined whether the targeted social information was lower or higher than *E_1_*. This approach using controlled social information is designed to measure individuals' propensity for social information use [39], but is less suitable for studying how social information may impact decision accuracy [43].

A participant's social information use (*s*) in a round is calculated as their relative estimate adjustment towards social information: *s* = (*E*_2_ − *E*_1_)/(*X* − *E*_1_). Rearranging the terms makes clear that *E*_2_ is an average of *E_1_* and *X*, weighted by *s*: *E*_2_ = (1 − *s*) × *E*_1_ + *s* × *X*. In other words, s indicates the relative weight assigned to social information. We characterized a participant's social information use as their adjustment *s* averaged across the five rounds of the task. In our analyses, we focus on cases 0 ≤ *s* ≤ 1, that is, where second estimates were a weighted average of *E_1_* and *X* [39]. Cases outside this range were removed from further analysis.

Participants were rewarded for accuracy. If an estimate was exactly correct, earnings were 100 points. For each animal the participant was off, we subtracted five points (in this task, 100 points equalled €0.30). This set-up ensures that participants should only use social information to adjust their first estimate if they believed it increased accuracy [39]. To avoid that participants would learn about their own performance or the usefulness of social information, they did not receive any feedback about their performance during the task.

### (c) Prosociality

**Figure 3.**
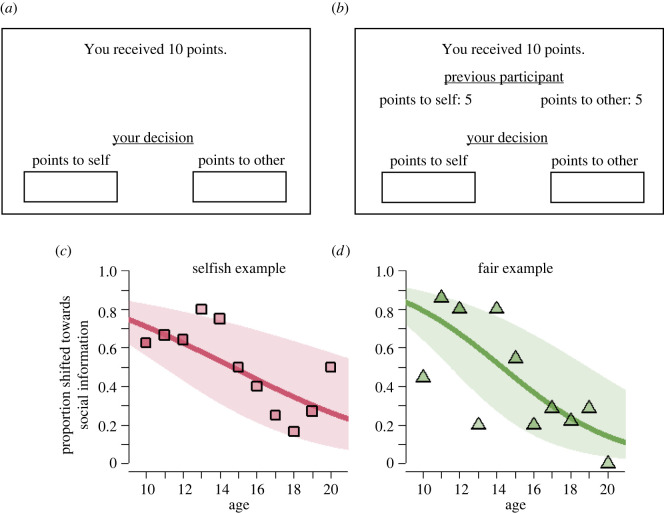
Social influence in prosociality decreases during adolescence. (*a,b*) Experimental task measuring prosocial behaviour with an adapted version of the dictator game. (*a*) Participants had to distribute 10 points between themselves and another participant. First, participants make this distribution decision alone. (*b*) Then, participants were matched with another participant, and made a second distribution decision upon observing how another (third) previous participant divided their 10 points. (*c,d*) Proportions of participants of adjusting donations toward social information as a function of age. (*c*) Adjustments when observing a peer who donated nothing (selfish example). (*d*) Adjustments when observing a peer who donated half their endowment (fair example). Lines show regression predictions, with standard errors in shades (see 'Methods'). For numbers of observations per data point, table 1. (Online version in colour.)

To measure peer effects in prosociality, we administered an adapted version of the well-studied dictator game (figure 3) [44]. Participants received an endowment of 10 points (worth €1.00) to divide between themselves and another person, who participated in a previous session. Only whole numbers were allowed, and the allocations needed to sum up to 10 for a participant to proceed. The decision situation minimizes the scope for strategic behaviour (e.g. due to reciprocity), so that any positive donation can be interpreted as a prosocial choice. Because of its clear and simple set-up, the task is suitable to study prosociality in developmental samples [45–47].

In the task, participants make two decisions. First, participants made a donation decision alone (*D*_1_; cf. figure 3*a*). Participants were then matched with another previous participant, and made a second decision (*D*_2_; cf. figure 3*b*). Before they made this second decision, they observed social information (*D_s_*) showing how another (third) previous participant (the ‘peer’) divided their 10 points. Then, they made a second donation decision (*D*_2_). We quantify social information use as (i) whether the participant moved towards the observed social information and (ii) the relative extent of adjustment towards social information calculated as (*D_2_* − *D_1_*)/(*D_S_* − *D_1_*).

In a within-subject treatment manipulation—administered in two different sessions—we varied the value of *D_S_*. In the ‘selfish peer’ condition, we showed as social information the donation of a previous participant who chose to keep all 10 points to themselves (i.e. *D_S_* = 0). In the ‘fair peer’ condition, we showed a previous participant who split the points equally (i.e. *D_S_* = 5).

In the associated analyses, we only included participants whose initial decision was not identical to social information (so that any adjustments towards social information were actually possible) or moved away from social information. That is, in figure 3*c*, we included participants who initially donated more than 0 (66%; 96 participants); and in figure 3*d*, we included participants who initially donated less than 5 (65%; 95 participants; for full details about numbers of participants in the analyses in each of the experiments, table 1).

### (d) Domain generality

Our within-subject implementation of the three tasks allowed us to test the domain generality of social information use. We conducted a confirmatory factor analysis on three variables characterizing participants' social information use in each of the three tasks, using the ‘lavaan’ package [48]. For calculating a factor characterizing social information use across domains for each participant, we required a measure of social information use in each of the three tasks (rule following: did or did not follow the examples; belief formation: average adjustment towards social information in the BEAST; and prosociality: an initial donation different from the social information in at least one of the conditions).

In the rule compliance task, we used a binary variable indicating whether or not a participant followed the examples (0 = stuck with initial behaviour; 1 = conformed to opposite social information). For the belief formation task (BEAST), we used the mean relative adjustment as outlined above. For the dictator game, we exploited all variation present in our data by calculating for both conditions the participants' relative adjustment towards social information (as for the BEAST; see above); if for a participant both were available (which was the case when in both conditions, initial donations were different from social information; see section 2c), we averaged the two.

**Figure 4.**
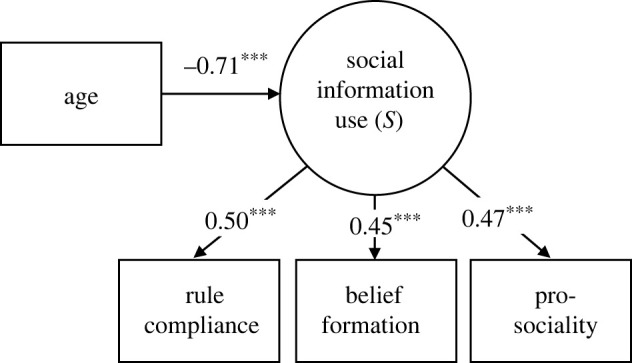
Decreases in social information use during adolescence reflect a domain-general pattern. Confirmatory factor analysis testing whether the covariance in measures from the three experiments is due to a single common factor of social information use (*S*). Numbers show standardized factor loadings, asterisks indicate significance levels (***p < 0.001; model fit: $\chi_{2}^{\;\;2}=1.386;$ *p* = 0.500; SRMSR = 0.044; see 'Methods' for details). See electronic supplementary material, table S5 for full model output.

Figure 4 summarizes the standardized solution of the latent variable model. The reported *χ*^2^ assesses the overall model fit and the discrepancy between the sample and fitted covariance matrices. The associated *p*-value of 0.500 indicates that we cannot reject the null hypothesis that the model predictions and the data are equal. The standardized root mean square residual (SRMSR) quantifies the difference between the residuals of the sample covariance matrix and the hypothesized model.

### (e) Statistical analyses

All statistical analyses were conducted in R v. 4.0.2 [49]. In figures 1–4, dot opacity reflects the number of participants underlying each dot; the numbers of participants in each age bin (i.e. each dot) for each experimental condition are given in table 1. Lines and shades in figures 1–3 show predictions of regressions fitted to participants’ behaviour, using ‘age’ as the sole predictor (see electronic supplementary material, tables S1–S3 for full models). Dependent variables were decisions to follow social information (figure 1*d,e*; binomial model), mean adjustment towards social information (figure 2*d*; linear model), decisions to move towards social information (figure 3*c,d*; binomial model), and the factor *S* (figure 4; linear model). Due to the novelty of some of the tasks, it was not possible to produce a proper *a priori* power analysis. Our sample size was therefore based on reported sample sizes in recent experimental work on social influence in adolescents [21,27,50,51].

## 3. Results

### (a) Rule compliance

Figure 1 outlines the traffic light task and its main results. When participants first completed the task on their own, 53% (77 out of 146) complied with the rule, foregoing about €0.50 in possible earnings. This compliance rate is comparable to previous findings with this paradigm from a large adult sample (58%) [38]. We observe no age trends in this initial behaviour (electronic supplementary material, figure S1; GLM: *β* = −0.032, 95% CI [−0.135, 0.072], *p* = 0.550; electronic supplementary material, table S1, model 1).

Peer effects in rule compliance markedly declined with age (figure 1*d,e*; GLM: *β* = −0.232, 95% CI [−0.354, −0.110], *p* < 0.001; electronic supplementary material, table S1, model 2). Overall, when observing three bad examples, 61% of participants violated the rule after having complied first (figure 1*d*). A substantially smaller portion (29%) of participants who initially violated the rule chose to comply upon observing three good examples (figure 1*e*; GLM: *β* = 1.379, 95% CI [0.629, 2.130], *p* < 0.001; electronic supplementary material, table S1, model 2). We did not find a significant interaction effect between age and condition (GLM: *β* =−0.137, 95% CI [−0.381, 0.108], *p* = 0.275; electronic supplementary material, table S1, model 3).

Fifty-five per cent (37 out of 67) of participants who followed the peers stuck with their updated behaviour in a third iteration of the task in which they completed the task alone again, suggesting that peers had some lasting effect on compliance. Sticking with updated behaviour did not appear to depend on participants' age or peer behaviour (GLM: *β* = −0.146, 95% CI [−0.320, 0.027], *p* = 0.099; electronic supplementary material, table S1, model 4). These results suggest that adolescents are not particularly unruly *per se* and good examples can promote compliance, but it also appears that early adolescents are easily swayed by their peers to change their behaviour and to break a rule.

### (b) Belief formation

Figure 2 summarizes the perceptual judgement task and its main results. In the overwhelming majority of cases (94%), revised estimates were weighted averages of initial estimates and social information (electronic supplementary material, figure S2). On average, revised estimates were closer to the true value than initial estimates (paired *t*-test: *t* = 17.06; d.f. = 729, *p* < 0.001), which is not surprising given that social information always pointed in the right direction.

Again, social information use substantially decreased across adolescence (figure 2*d*; LMM: *β* = −0.021, 95% CI [−0.032, −0.011], *p* < 0.001; electronic supplementary material table S2). Ten-year-olds on average assigned about equal weight to their own first estimate and social information (*y* ≈ 0.5 in figure 2*d*). Twenty-year-olds on average assigned four times less weight to social information than to own estimates (*y* ≈ 0.2). These results reveal how ‘egocentric discounting’, a commonly observed phenomenon in social information use in adults [39,42,52,53], emerges during adolescence.

### (c) Prosociality

Figure 3 shows the decision situation in the dictator game and summarizes its main results. Participants first completed the task on their own. Mean initial donations across both sessions were 2.7 (s.d. = 2.3), and did not significantly differ across age (in line with [54–56]; LMM: *β* = −0.069, 95% CI [−0.164, 0.025], *p* = 0.150; electronic supplementary material, table S3, model 1).

Peer effects on donations were substantial (figure 3*c,d*; electronic supplementary material, figure S3). After observing a selfish example, 46% of participants who initially donated more than 0 adjusted their donation downwards, reducing average donations by 0.89 (95% CI [−1.19, −0.59]). After observing a fair example, 50% of participants who initially donated less than 5 adjusted their donation upwards, increasing average donations by 0.95 (95% CI [0.64, 1.25]; paired t-tests comparing initial and revised donations, selfish example: *t* = −5.816, d.f. = 145, *p* < 0.001; fair example: *t* = 5.988, d.f. = 145, *p* < 0.001).

Peer effects in prosociality strongly decreased with age: older adolescents were less likely to adjust their donations towards those of the peer (figure 3*c,d*; logistic GLMM: *β* =−0.269, 95% CI [−0.376, −0.161], *p* < 0.001; electronic supplementary material, table S3, model 2). The magnitude of donation adjustments also decreased with age (logistic GLMM: *β* = −0.136, 95% CI [−0.203, −0.070], *p* < 0.001; electronic supplementary material, table S3, model 3), and did not differ between conditions (same model: *β* = 0.001, 95% CI [−0.361, 0.364], *p* = 0.995; electronic supplementary material, table S3, model 3). These results indicate that both selfish and fair examples substantially modulate altruistic giving, and that their impact on behaviour steadily decreases during adolescence.

### (d) Domain generality

In each of our tasks, we observe a downward trend in social information use across age. This suggests that there may be a common mechanism driving peer effects across domains. Our experiments allow us to test that idea. Indeed, tendencies to use social information were positively correlated across the three experiments (electronic supplementary material, table S4). To further examine the underlying structure of the data shown in figures 1–3, we conducted a confirmatory factor analysis. This analysis supports the idea that the observed peer effects in each behavioural domain reflect a single underlying factor of social information use (figure 4; electronic supplementary material, table S5). This factor—which we dub *S*—steadily declines during adolescence (figure 4; effect of age: *β* = −0.713, 95% CI [−0.922, −0.503], *p* < 0.001; electronic supplementary material, table S5).

## 4. Discussion

Our paper can be summarized in three main points. First, we present an integrated set of incentivized experiments showing how social environments can negatively and positively impact adolescents' behaviour across different key domains of behaviour, including rule compliance and belief formation, which have hitherto been understudied in adolescence. In rule compliance, the effects of bad examples induce much disobedience, but good examples also substantially increase rule compliance. Peers also have a pronounced effect on belief formation in a setting where social values and preferences are irrelevant. Prosocial behaviour is similarly increased or decreased by exposure to good (fair-sharing) or bad (selfish) examples. Second, we show that across each of these domains, the potential of peers to impact behaviour steadily decreases throughout adolescence. Third, our results suggest that the decreasing impact of peers across adolescence reflects a developmental decline in a domain-general factor of social information use.

Our three experiments consistently show that social information use declines across adolescence (figures 1–4). Young adolescents have a high sensitivity to peer behaviour, while older adolescents prioritized personal preferences and beliefs (reinforcing results of [19,20,34,57]). In the context of rule compliance and prosociality, the marked impact of social information among early adolescents might reflect high levels of uncertainty about their personal values [8], that is, they might have a less clear notion of what is the right thing to do. Older adolescents might be less uncertain of these values, and reduced uncertainty might weaken the impact of social information on behaviour [58,59]. This uncertainty about values thus makes adolescents more sensitive to social information, and makes it easier for new social values to emerge during this developmental phase. As such, adolescents may become a driving force in the evolution of our cultural values (e.g. norms around gun ownership, or environmental issues).

However, the results of our belief formation task (figure 2) indicate that uncertainty about values cannot be the whole story. In this task, values and preferences were irrelevant but resulted in the same developmental pattern of diminishing use of social information across adolescence. Our results suggest the existence of a domain-general factor of social information use *S*, which is very high in early adolescence and decreases as people approach adulthood. This finding provides support for the implicit assumption in previous research that developmental trends in social information use would generalize across different domains of behaviour, and would shape behaviour in positive and negative directions in similar ways [12,60]. Future experimental and field studies could test the reliability and robustness of *S* beyond the three domains studied in this paper, examine its links with related constructs, such as social competence and social attention [61–63], and investigate contextual effects (e.g. of being observed by others [64], a factor that can modulate peer influence in adolescents [5,59,65]). Such studies would help strengthen the empirical foundation of this factor, delineate its psychometric structure and its significance beyond laboratory settings [66]. We believe such research would contribute to a comprehensive and more mechanistic understanding of social influence across adolescence.

One might suspect that the reported developmental trends in susceptibility for social influence (*S*) are due to decreasing stochasticity in decision making with age [51], that is, younger participants might behave more randomly. We believe that stochasticity does not play an important role in the reported age effects. In our rule-following task, younger participants were not more likely to switch back after updating their behaviour (electronic supplementary material, table S1, model 4). Moreover, increased stochasticity in the belief formation task would not lead to more adjustments towards social information, but to more adjustments in any direction. Similarly, in the prosociality task, randomness would not systematically bias adjustments in the direction of social information. This suggests that the decreasing trend in susceptibility for social influence (*S*) cannot be explained by decreased stochasticity.

Our experiments tightly controlled the provision of social information, allowing us to quantify how good and bad examples impacted behaviour, while avoiding confounding effects of individuals influencing each other, and selecting certain people as social sources [67]. In real life, however, the social information that might influence individuals depends on their position in their social network, and individuals’ social learning strategies (e.g. to preferentially heed familiar individuals, experts or prestigious sources [68–72]). This introduces asymmetries in the social environments that individuals observe, determining, for example, whether rule compliance and generosity are perceived to be common, and whether rule violations and selfishness are typically disapproved of [38,71,73]. Exploring the interplay between behaviour, social learning, and network formation is key for understanding how peers can promote and consolidate positive behaviour, and why people end up running in the wrong crowd in which socially undesirable conduct is exacerbated.

As their privileges grow, adolescents have to figure out how to relate to myriad novel social situations across many different domains of behaviour. Our results show that across domains, the behaviour of early adolescents is strongly guided by the social environment. They suggest that peer influence is a double-edged sword: it can provoke rule violations and reduce prosociality, but can also prompt rule compliance, promote prosociality, and facilitate the integration of social information to form beliefs about the world. These insights can inform the timing and design of interventions emphasizing positive examples in adolescents' social networks [74]. While many interventions are based on building individuals’ resistance to peer influence [57,75], our study highlights the potential of peers to spread prosocial norms. Both offline and on social media platforms, enhancing the salience of good examples and well-informed peers may bias norm perceptions in socially desirable ways and boost the formation of accurate beliefs. This way, social influence among adolescents can be harnessed to positively impact developmental trajectories, reducing anti-social behaviour and false beliefs, while facilitating individuals' attunement to their social environment and promoting socially desirable outcomes.

## Data Availability

All data and code associated with this work are available from the public repository, accessible via https://github.com/LucasMolleman/Social_influence_in_adolescence. Experimental code is available (in editable form) on the LIONESS Lab platform (https://lioness-lab.org). Electronic supplementary material is available online [76].
